# Supplementary material for: An Epigenomic fingerprint of human cancers by landscape interrogation of super enhancers at the constituent level
Source: PLoS Comput Biol. 2024 Feb 9;20(2):e1011873. doi: 10.1371/journal.pcbi.1011873 (PMC10883583; doi:10.1371/journal.pcbi.1011873)
Supplement: S1 Fig — Dashed line indicates 25% overlap cutoff. a. SE median width b. Total number of SEs c. CE median width d. Total number of CEs. (PDF) [file pcbi.1011873.s001.pdf]

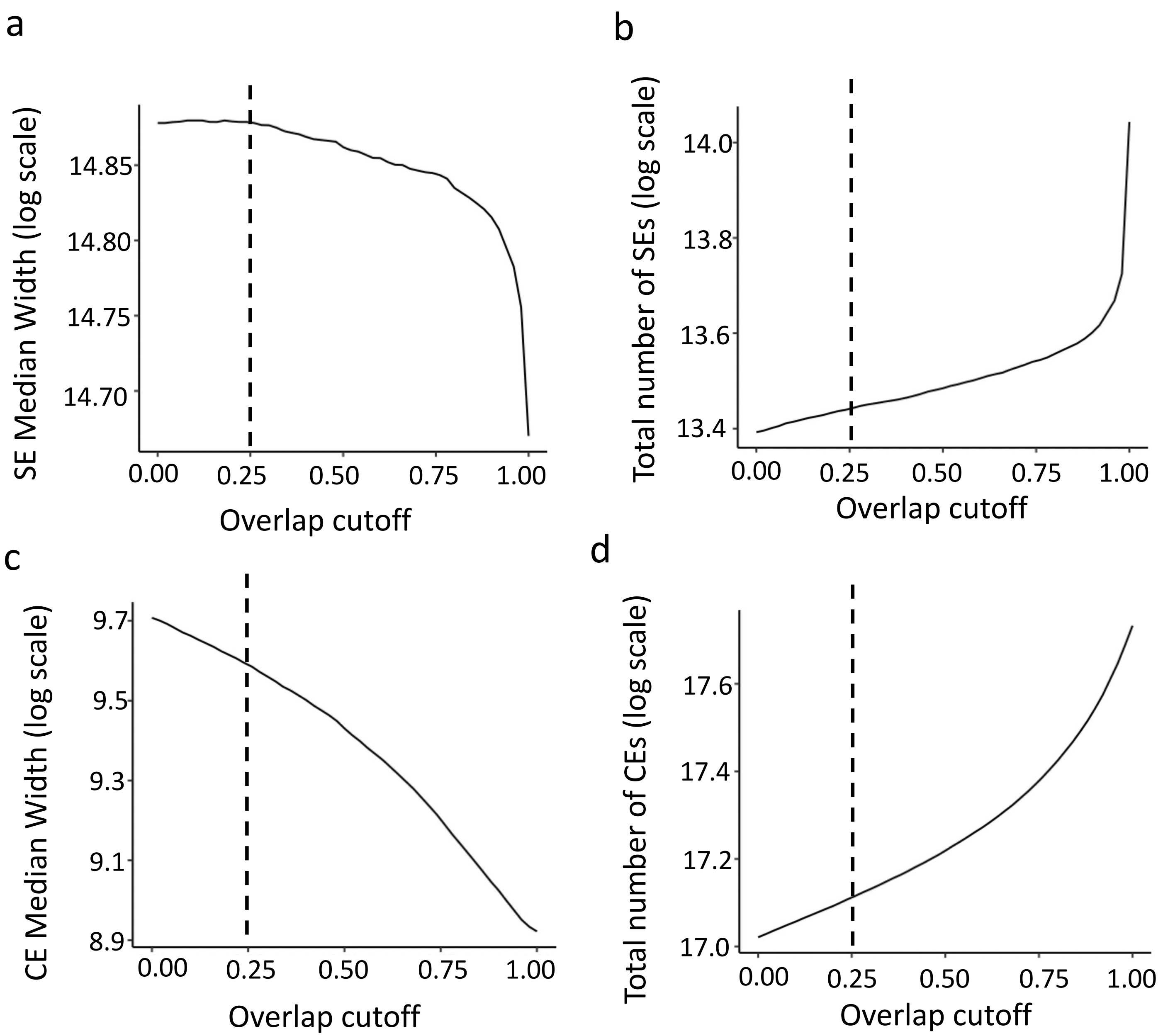

S1 Fig. Median width and total number of merged SEs and CEs based on different overlap cutoffs. Dashed line indicates 25% overlap cutoff. **a.** SE median width **b.** Total number of SEs **c.** CE median width **d. T**otal number of CEs.
