# Supplementary material for: An Epigenomic fingerprint of human cancers by landscape interrogation of super enhancers at the constituent level
Source: PLoS Comput Biol. 2024 Feb 9;20(2):e1011873. doi: 10.1371/journal.pcbi.1011873 (PMC10883583; doi:10.1371/journal.pcbi.1011873)
Supplement: S2 Fig — Data points indicate the estimated means of the lower and higher mixtures in individual cell lines. (PDF) [file pcbi.1011873.s002.pdf]

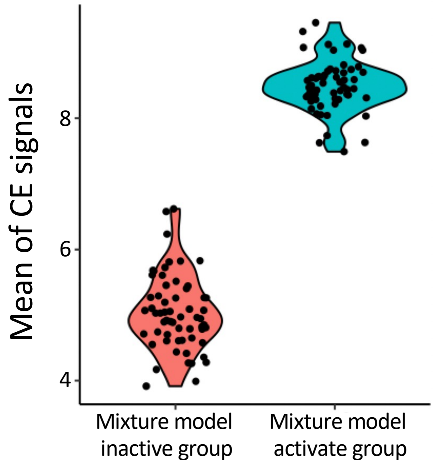

S2 Fig. Genome-wide priors of the inactive and active enhancer groups estimated across 60 cancer cell lines. Data points indicate the estimated means of the lower and higher mixtures in individual cell lines.
