# Supplementary figures and images for: An Epigenomic fingerprint of human cancers by landscape interrogation of super enhancers at the constituent level
Source: PLoS Comput Biol. 2024 Feb 9;20(2):e1011873. doi: 10.1371/journal.pcbi.1011873 (PMC10883583; doi:10.1371/journal.pcbi.1011873)

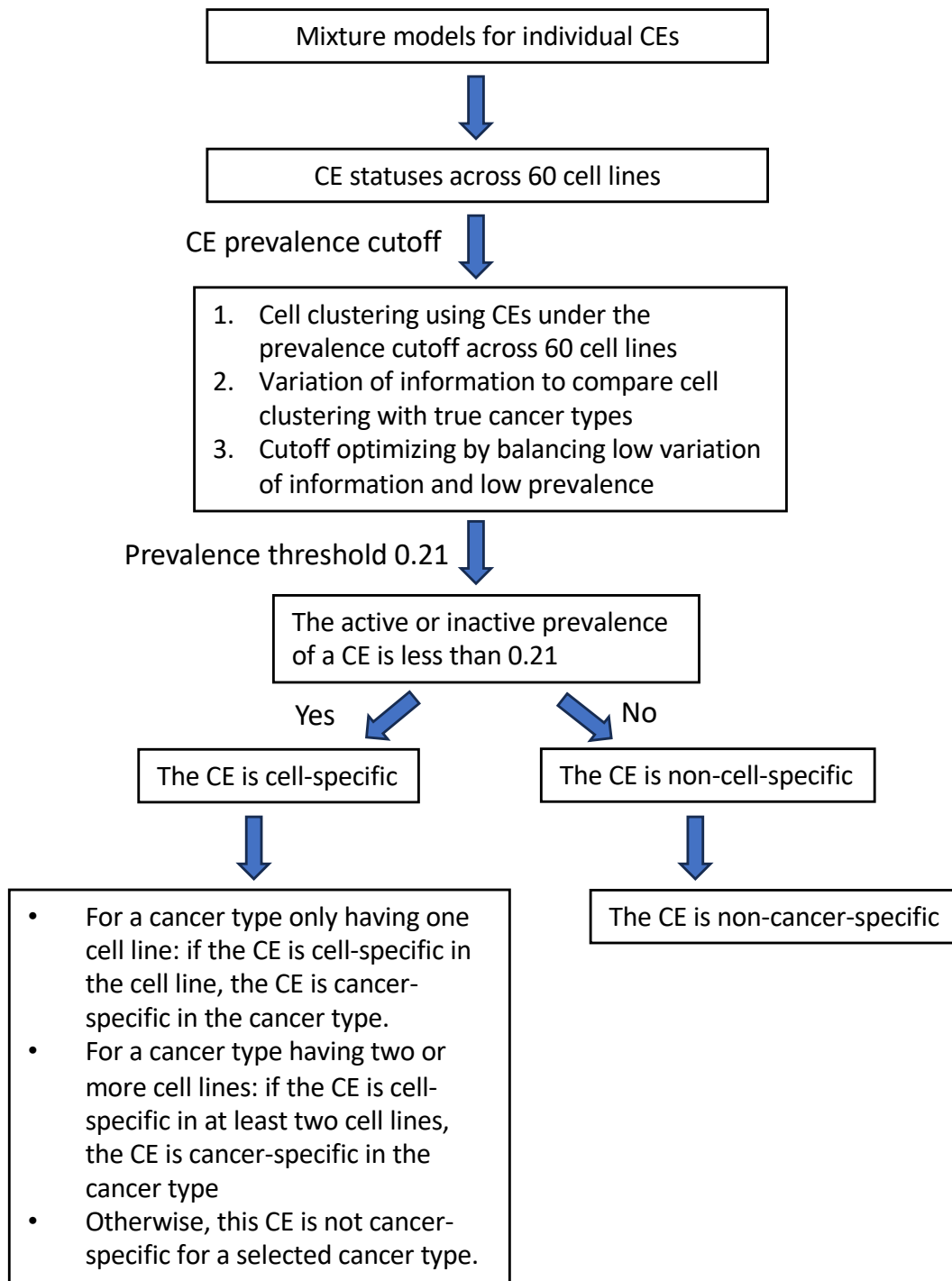

**S3 Fig. Flowchart to define cell/cancer-specific CEs.**

Supplement: S3 Fig — (PDF) [file pcbi.1011873.s003.pdf]

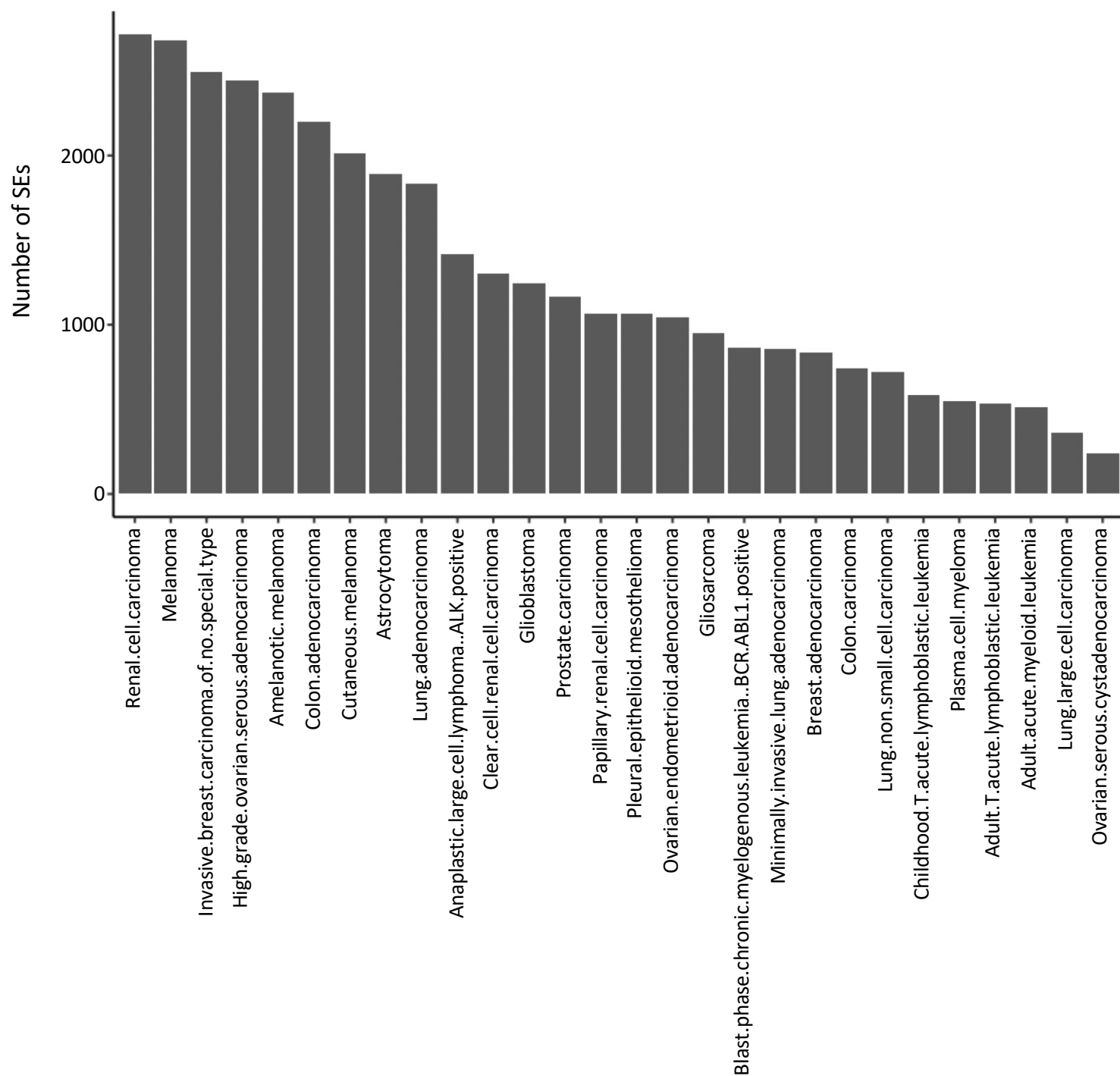

S4 Fig. Number of SEs identified in different cancers.

Supplement: S4 Fig — (PDF) [file pcbi.1011873.s004.pdf]

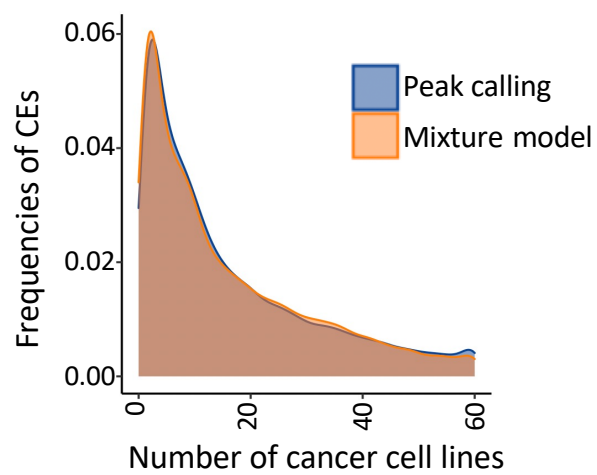

**S6 Fig. Overall prevalence of active CEs identified by mixture models and peak calling.**

Supplement: S6 Fig — (PDF) [file pcbi.1011873.s006.pdf]

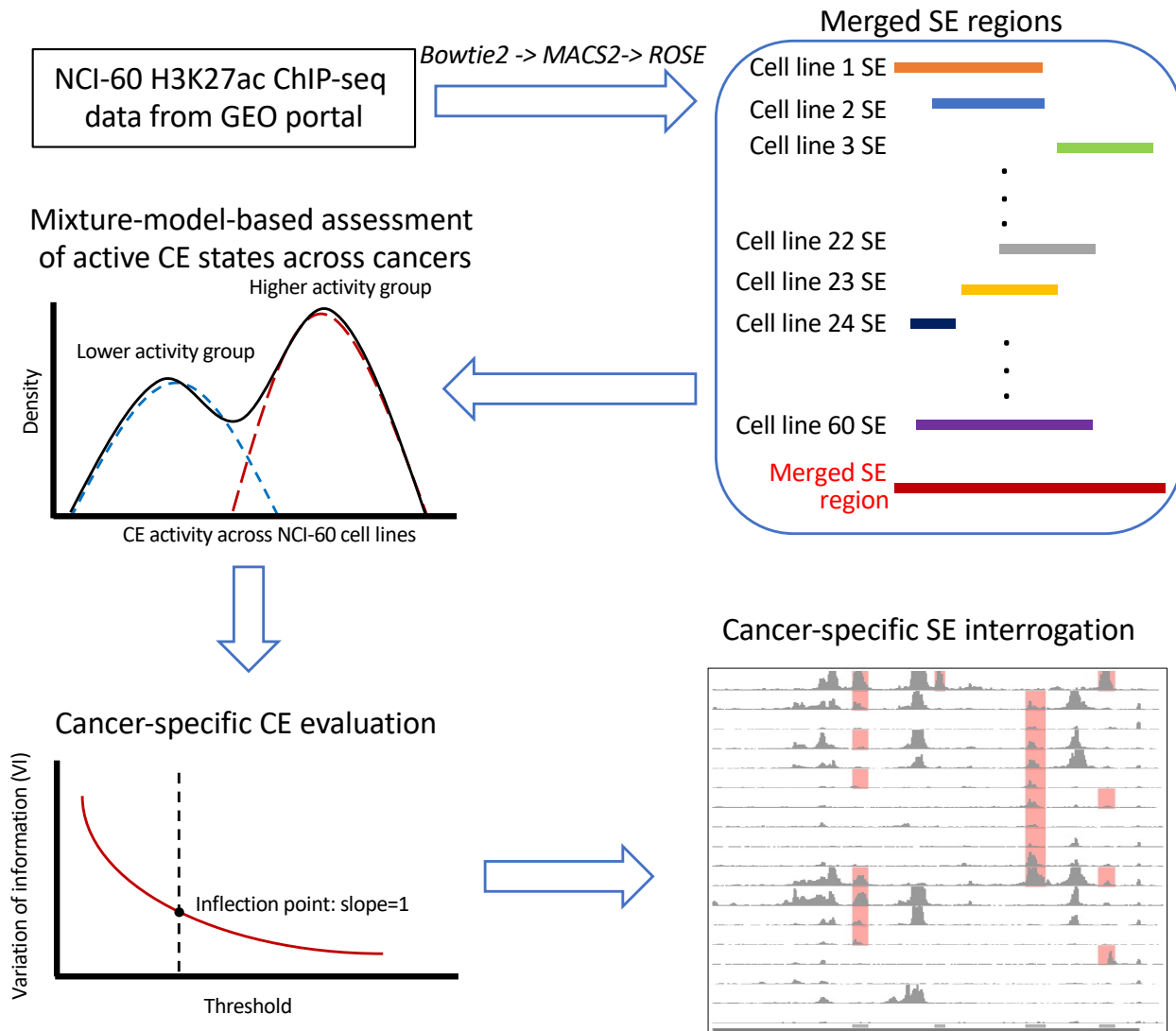

**S11 Fig. Flowchart of identifying fingerprint SE signatures across cancers.**

Supplement: S11 Fig — (PDF) [file pcbi.1011873.s011.pdf]
