# Supplementary material for: An Epigenomic fingerprint of human cancers by landscape interrogation of super enhancers at the constituent level
Source: PLoS Comput Biol. 2024 Feb 9;20(2):e1011873. doi: 10.1371/journal.pcbi.1011873 (PMC10883583; doi:10.1371/journal.pcbi.1011873)
Supplement: S5 Fig — Left: normalized ChIP-seq signals recovers enhancer activity in MDA-MB-231 cell line; right: raw signals indicate no active enhancers at the same location due to low ChIP-seq coverage in MDA-MB-231. (PDF) [file pcbi.1011873.s005.pdf]

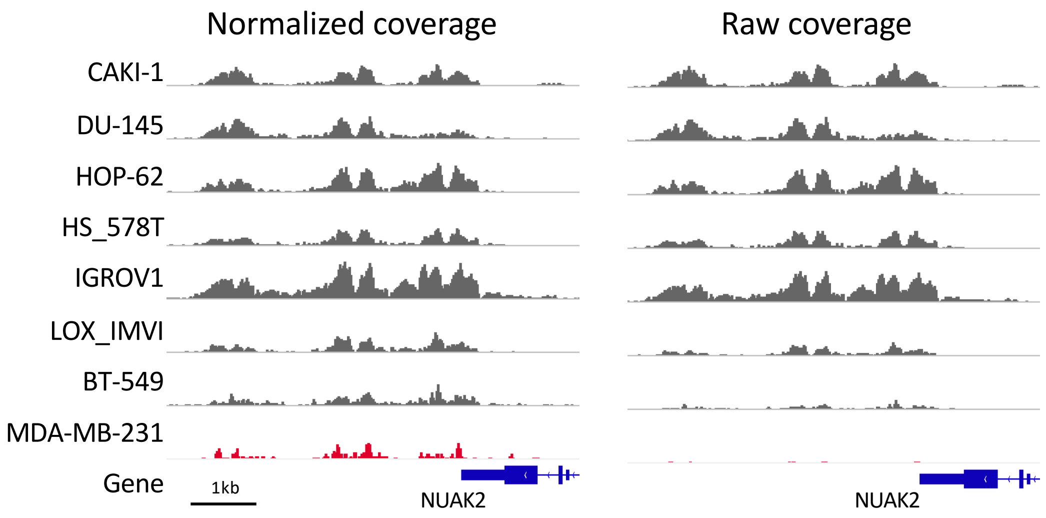

S5 Fig. Vertical comparison of H3K27Ac signals across cancers refines enhancer activity consensus in individual samples. Left: normalized ChIP-seq signals recovers enhancer activity in MDA-MB-231 cell line; right: raw signals indicate no active enhancers at the same location due to low ChIP-seq coverage in MDA-MB-231.
