# Supplementary material for: An Epigenomic fingerprint of human cancers by landscape interrogation of super enhancers at the constituent level
Source: PLoS Comput Biol. 2024 Feb 9;20(2):e1011873. doi: 10.1371/journal.pcbi.1011873 (PMC10883583; doi:10.1371/journal.pcbi.1011873)
Supplement: S7 Fig — (a). Red shaded square is an enhancer region under-estimated in A549 cell line by peak calling methods but identified as active by mixture model. This region shows strong enhancer activity in A549 compared to other cancer cell lines and presents regulatory interactions with target gene TRMT5 based on ChIA-PET data. (b). Similar to a) but for another enhancer region regulating SIX4 gene in A549 cell line. (c). Similar to a) but for another enhancer region regulating GNAI3 gene in HCT-116 cell line. (d). Similar to a) but for another enhancer region regulating HID1, MRPL58 and KCTD2 in HCT-116 cell line. (PDF) [file pcbi.1011873.s007.pdf]

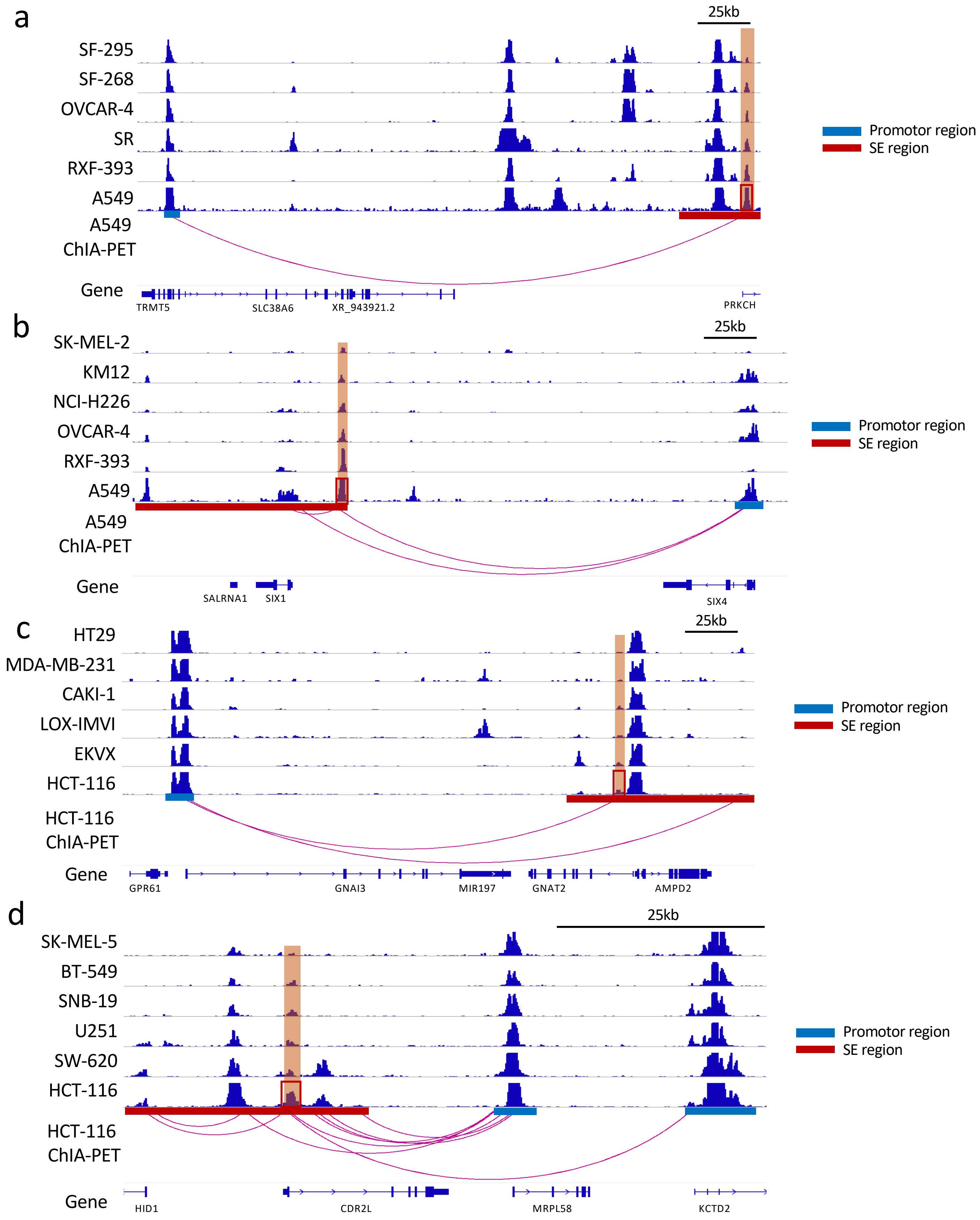

S7 Fig. Extra examples illustrating the improved sensitivity in detecting true active CEs by mixture models compared to peak calling. (a). Red shaded square is an enhancer region under-estimated in A549 cell line by peak calling methods but identified as active by mixture model. This region shows strong enhancer activity in A549 compared to other cancer cell lines and presents regulatory interactions with target gene TRMT5 based on ChIA-PET data. (b). Similar to a) but for another enhancer region regulating SIX4 gene in A549 cell line. (c). Similar to a) but for another enhancer region regulating GNAI3 gene in HCT-116 cell line. (d). Similar to a) but for another enhancer region regulating HID1, MRPL58 and KCTD2 in HCT-116 cell line.
