# Supplementary material for: An Epigenomic fingerprint of human cancers by landscape interrogation of super enhancers at the constituent level
Source: PLoS Comput Biol. 2024 Feb 9;20(2):e1011873. doi: 10.1371/journal.pcbi.1011873 (PMC10883583; doi:10.1371/journal.pcbi.1011873)
Supplement: S8 Fig — Blue shaded square is an enhancer region over-estimated in A549 cell line by peak calling methods but identified as inactive by mixture model. This region shows weak enhancer activity in A549 compared to other cancer cell lines and presents no regulatory interactions with any genes based on ChIA-PET data. (b). Similar to a) but for another enhancer region in A549 cell line. (c). Similar to a) but for another enhancer region in HCT-116 cell line. (d). Similar to a) but for another enhancer region in MCF7 cell line. (PDF) [file pcbi.1011873.s008.pdf]

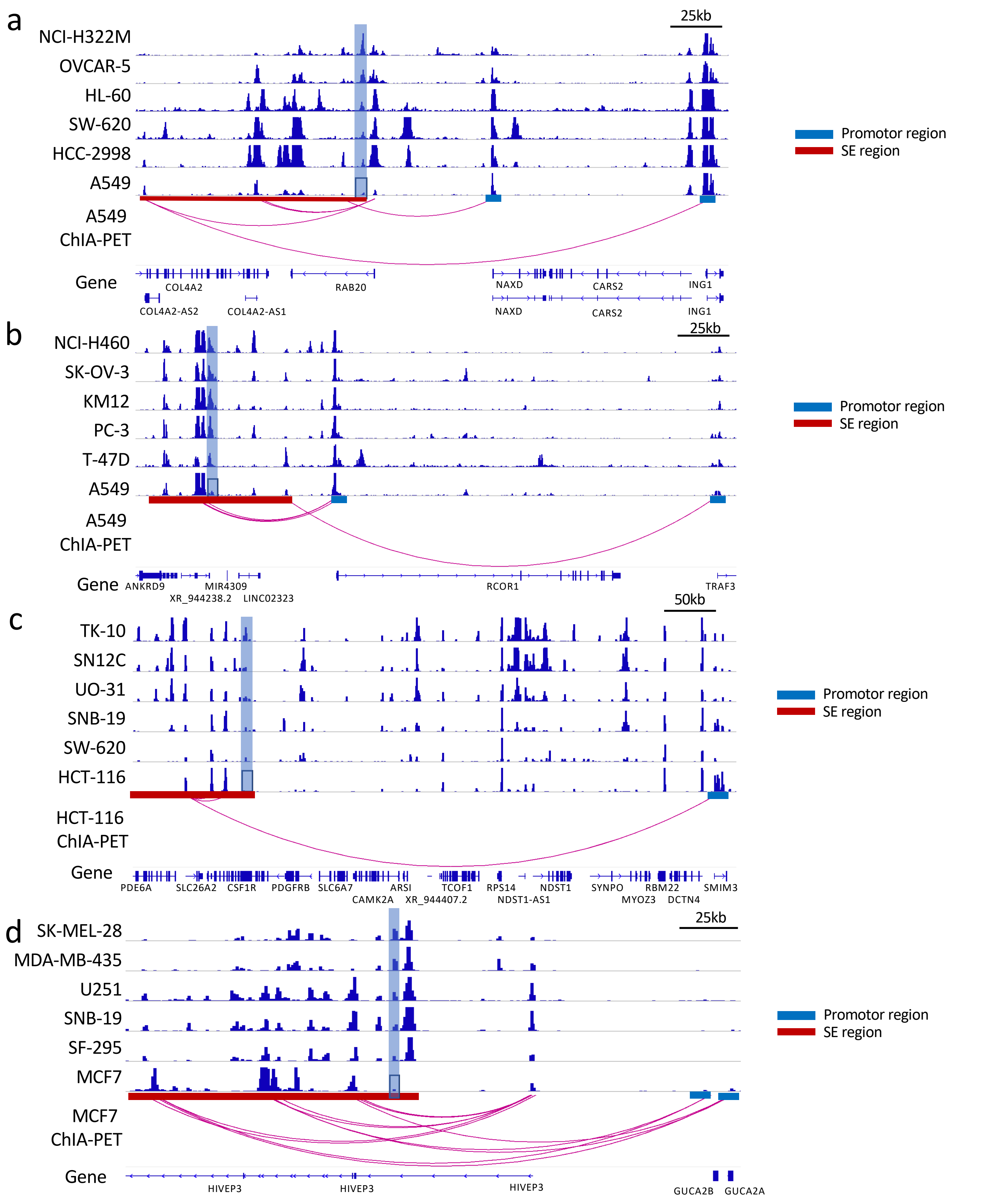

S8 Fig. Extra examples illustrating the improved specificity in detecting true inactive CEs by mixture models compared to peak calling. Blue shaded square is an enhancer region over-estimated in A549 cell line by peak calling methods but identified as inactive by mixture model. This region shows weak enhancer activity in A549 compared to other cancer cell lines and presents no regulatory interactions with any genes based on ChIA-PET data. (b). Similar to a) but for another enhancer region in A549 cell line. (c). Similar to a) but for another enhancer region in HCT-116 cell line. (d). Similar to a) but for another enhancer region in MCF7 cell line.
