# Supplementary material for: An Epigenomic fingerprint of human cancers by landscape interrogation of super enhancers at the constituent level
Source: PLoS Comput Biol. 2024 Feb 9;20(2):e1011873. doi: 10.1371/journal.pcbi.1011873 (PMC10883583; doi:10.1371/journal.pcbi.1011873)
Supplement: S9 Fig — (PDF) [file pcbi.1011873.s009.pdf]

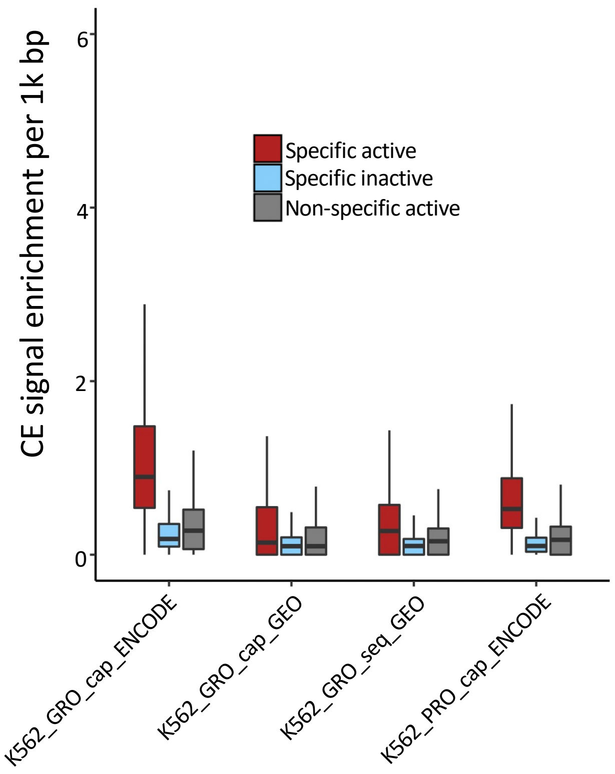

S9 Fig. Enhancer activity from extra nascent RNA sequencing datasets for K562, including GRO-cap, Pro-cap and GRO-seq, from the ENCODE and GEO data repositories.
