# Supplementary material for: An Epigenomic fingerprint of human cancers by landscape interrogation of super enhancers at the constituent level
Source: PLoS Comput Biol. 2024 Feb 9;20(2):e1011873. doi: 10.1371/journal.pcbi.1011873 (PMC10883583; doi:10.1371/journal.pcbi.1011873)
Supplement: S10 Fig — a. STARR-seq. b. PRO-seq. (PDF) [file pcbi.1011873.s010.pdf]

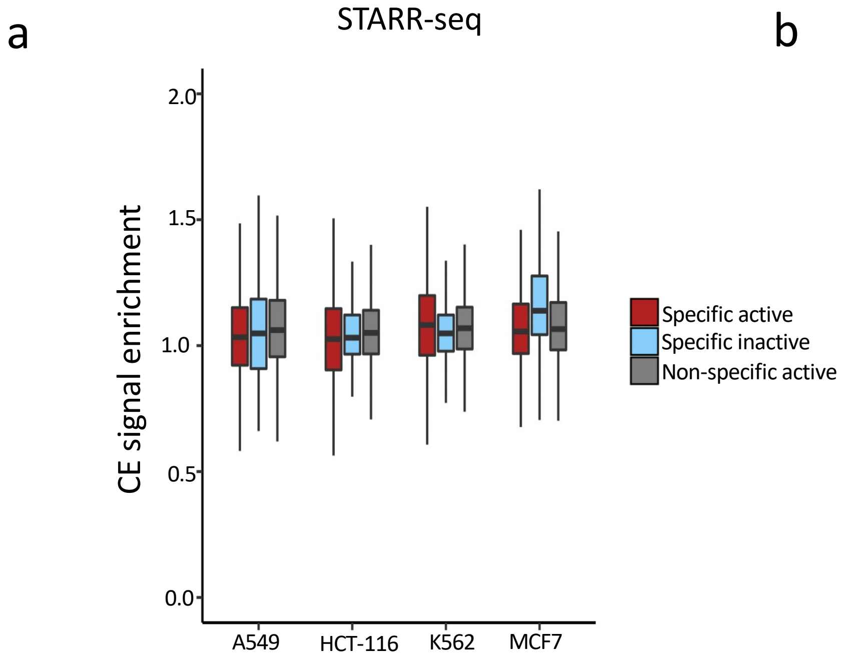

S10 Fig. STARR-seq and PRO-seq based CE activity without normalization of CE width. a. STARR-seq. b. PRO-seq
