# Supplementary material for: An Epigenomic fingerprint of human cancers by landscape interrogation of super enhancers at the constituent level
Source: PLoS Comput Biol. 2024 Feb 9;20(2):e1011873. doi: 10.1371/journal.pcbi.1011873 (PMC10883583; doi:10.1371/journal.pcbi.1011873)
Supplement: S12 Fig — Highlighted in blue square is a cell- specific active CE in K562 but inactive in A549. This CE links to promoters of two gene targets, ANKRD9 and RCOR1, suggesting its cell-specific regulation in K562. (PDF) [file pcbi.1011873.s012.pdf]

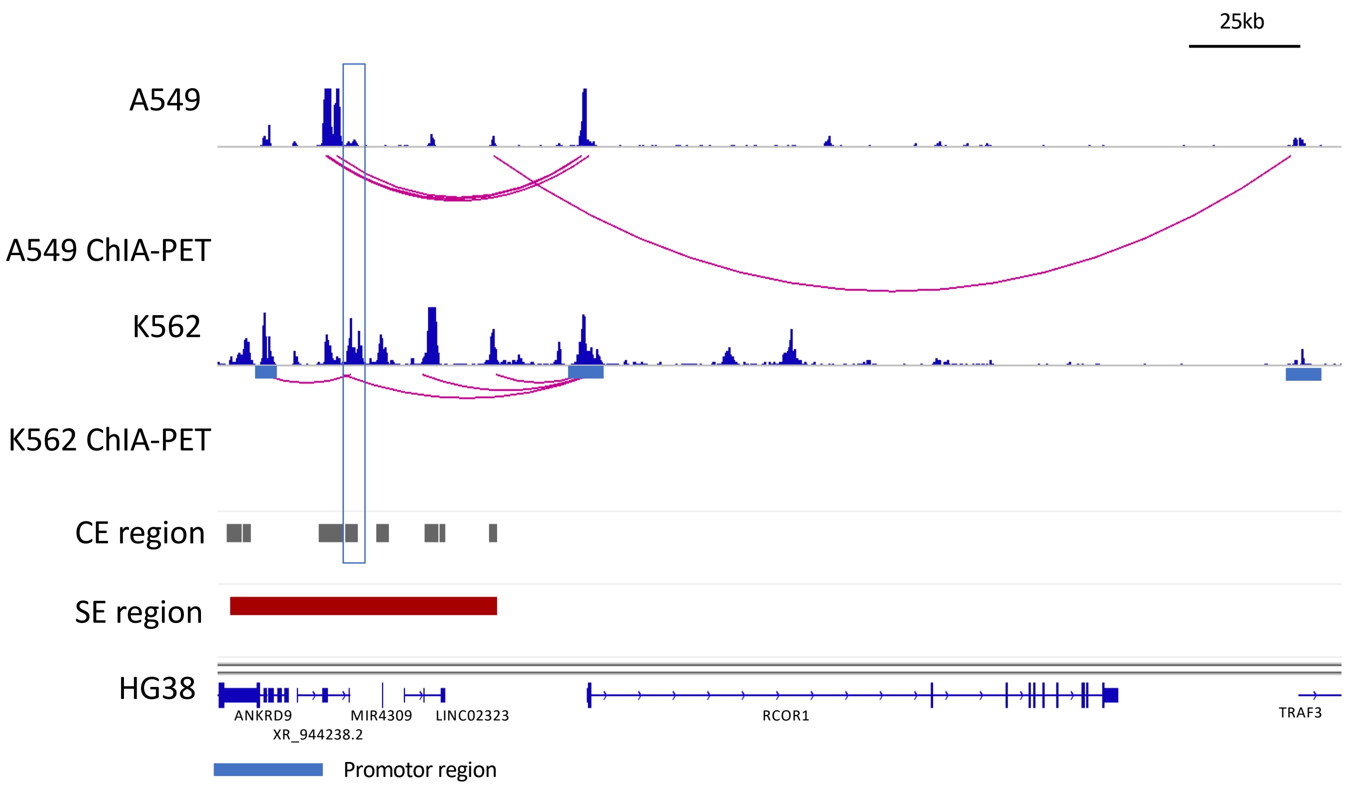

S12 Fig. An example of interpreting cancer-specific active CEs. Highlighted in blue square is a cell-specific active CE in K562 but inactive in A549. This CE links to promoters of two gene targets, *ANKRD9* and *RCOR1*, suggesting its cell-specific regulation in K562.
