# Supplementary material for: An Epigenomic fingerprint of human cancers by landscape interrogation of super enhancers at the constituent level
Source: PLoS Comput Biol. 2024 Feb 9;20(2):e1011873. doi: 10.1371/journal.pcbi.1011873 (PMC10883583; doi:10.1371/journal.pcbi.1011873)
Supplement: Attachment — Submitted filename: point-by-point response_final.docx [file pcbi.1011873.s015.docx]

*We thank the reviewers for their comments and suggestions which greatly help improve our manuscript.*

**Reviewer #1:**

In this manuscript, Liu et al. describe an advanced computation study focused on SEs’ structure and function in cancer. The authors applied bioinformatics analysis in diverse sets of publicly available datasets in order to decipher how SEs are structured and how their composites operate in-between diverse cancer tissue types. Overall, the study is well-designed and efficiently implemented. The authors managed to zoom into SEs entities at the constituent level, and this is an achievement. They applied straightforward criteria to distinguish active versus inactive states of SEs’ constituents, and to classify cell-type specific SEs. Finally, the authors developed a computational package that can be applied for the exploration of these findings. The manuscript is well-written.

*Response: We appreciate the reviewer for the overall positive comments.*

Major points

1. Page 11: “POLR2A ChIA-PET sequencing can identify chromatin interactions between enhancers and promoters, thus allows us to functionally evaluate CE activity in regulating target genes. Using public POLR2A ChIA-PET datasets of four cancer cell lines, we found that the active CEs only by the mixture models interacted more frequently with cis-regions (on average 3.4 interactions) and linked to more target genes (on average 5.5 genes), compared to those by peak calling only (on average 2.9 interactions and 3.3 genes, respectively) (Fig 3B-C).”

The fact that a cis-acting element develops 3D associations in vivo with another genomic loci, e.g. a promoter or a gene, does not necessarily equal to its increased capability for being active but elevates its potential for such a functional conductance. I believe that the authors have to complement this analysis by providing details relative to the transcriptional states of the interacting genes with the CEs identified. They have to analyze gene expression data and to demonstrate any correlation between the CEs and the genes that are detected in physical proximity according to POLR2A ChIA-PET.

*Response #1: This is truly a great suggestion. We added new analysis to demonstrate the correlation between active CEs and their targeted gene expression.*

*Specifically, for the four cell lines that have POLR2A ChIA-PET data available, we downloaded their gene expression data from DepMap data portal (1) (*[*https://depmap.org/portal*](https://depmap.org/portal)*), where gene expression profiles for 1450 cell lines are available. The gene expression values were transformed to percentile-based measurements for each gene to ease the comparison of gene expression states across genes. For each gene, the minimum of its expression across all 1450 cell lines was transferred to zero-percentile while the maximum was transferred to 100th percentile. In other words, higher percentile means the gene holds higher expression in a given cell line.*

*We then compared the expression states of genes targeted by the identified active CEs from our model, the peak-only method and both methods in the four selected cell lines. We found targeted genes by the active CEs from our model (either by our model only or by both our model and peak calling) showed higher expression levels compared to those by the peak-calling method only. This adds evidence that the CEs identified by our proposed model presented high activity. We add one sub-figure (see below) into the revised Figure 3 (also showed below) to demonstrate this.*

*Revised as recommended.*

2. Figure 3E: The panel demonstrates a promoter region that is located internally within the MIR1252 locus (bottom of the panel). Is this a cryptic promoter? The authors need to clarify. In addition, more examples analogous to those provided are required to support the findings described in this paragraph.

*Response #2: We apology for the confusion due to that we mislabeled the gene names related to that promoter. This isn’t a cryptic promoter. There are two genes overlapped at this genomic location: MIR1252 and SYT1, a protein coding gene. The promoter we highlighted actually was located close to the transcription start site of the SYT1 transcripts.*

*In the revised version of manuscript, we have added 8 more examples in the Supplemental Figures S7-S8 and as shown below. In addition, we slightly optimized our mixture model based on the suggestions from Reviewer 2. Now, we highlight another example in Figure 3F to avoid any confusion.*

*Revised as recommended.*

3. Figure 5A and B: The resolution of the figure should be improved. The color code is not displayed in high resolution and the size of the panels should be increased in order to facilitate the readers to follow the information incorporated.

*Response #3: We thank the review for the suggestion. We have revised the figure with improved resolution, color code and panel size. Revised as recommended.*

4. Page 12: The authors have to provide more details in the main text regarding how they assessed the STARR-seq datasets. In addition, they have to highlight that STARR-seq is a reporter-gene-based assay implemented by the construction of plasmid libraries that are introduced in cells.

*Response #4: We apology for the confusion. We have added more details of STARR-seq/Pro-seq data processing at the Methods section “Activity evaluation of the specific and non-specific CEs”. We also added description of STARR-seq in the revised Results, as the reviewer suggested and with proper citations added. Revised as recommended.*

Minor points

1. The authors utilize a spectrum of publicly available datasets. It will be helpful for the readers if the authors include the citations in the corresponding paragraphs of the results section.

*Response: We have added citations at the corresponding paragraphs of the Results section. Revised as recommended.*

2. “We generated cancer-specific SEs based on their CE statuses for all cancer types in the NCI-60 cell panel (Fig 5A).” The authors have to rephrase this sentence. I believe the verb “generate” is not the right one here.

*Response: We replaced “generated” with “summarized”. Revised as recommended.*

**Reviewer #2:**

This manuscript presents a comprehensive exploration of cancer-specific super enhancer (SE) signatures at the constituent level for 28 cancer types in the NCI-60 cell panel. The authors employ a Gaussian mixture model to distinguish active constituent enhancers (CEs) from inactive ones. Additionally, they develop an R package to facilitate data exploration and visualization. The manuscript is well written, with clear structure, and offers valuable contributions to the field. However, there are several concerns, primarily pertaining to the methodology and clarity in certain sections.

*Response: We appreciate the reviewer for the overall positive comments.*

Major:

1. The description of SE and CE candidate list creation lacks clarity, especially regarding the estimation of CEs. Further elaboration on the distinction between the estimation of SEs and CEs is necessary. Additionally, a rationale for the chosen percentage thresholds (20% width overlapping for SEs and 10% width overlapping for CEs) is needed, along with relevant references to support this choice.

*Response #1: We have added clarifications, supporting figures and references regarding the creation of candidates lists of SEs and CEs in the revised manuscript. In short, we have revised both numbers as 25% based on both reference suggestions and the rationale below. We found the candidate lists don’t change much, regarding their overall widths and numbers, when the overlap cutoffs alter within a controlled range (0 to 25%, figures below and Fig S1).*

*For SEs, we and others have previously demonstrated that their constituent alterations are common across cell conditions (2-4). To properly capture such constituent alterations, we need to increase the chances of comparisons within the same SEs and decrease the chances of comparisons between consecutive SEs. We set an overlap cutoff (based on the percentage of SE size) to merge consecutive SE regions across cell lines to balance the two needs. Consecutive SE regions with an overlap larger than the cutoff were merged. We observed that increased cutoffs generally cause decreased median width and increased total number of final SEs since shorter overlaps aren’t merged. We chose 25% as the threshold cutoff in the revised manuscript as cutoffs beyond 25% cause dramatic decreasing of SE widths, likely over-fragmenting regions of the same SE into different SEs. The total numbers of merged SEs vary less than 3.4% and median widths vary less than 0.2% when using different cutoffs within the range of 0~25%, suggesting minor effects of cutoff selection during merging SE regions.*

*CEs are much shorter, usually hundreds of base pairs, compared to SEs. Experimental and biological factors, such as sequencing fragment size selection and transcription factor motif distribution within CEs, could bias the precise identification of CE locations. An overlap cutoff to merge consecutive CEs helps make comparisons more robust to the bias effects. Previous publications applied the cutoff 25% as the minimum percentage of overlap to merge ChIP-seq peaks (5,6). We chose 25% in the revised manuscript following these publications. In addition, we observed that the widths and total number of merged CEs had limited variation (8.6% and 6.5%, respectively) when cutoffs change between 0 to 25%, also suggesting limited effects of cutoff selection within the range.*

2. The approach to bootstrapping raises concerns. Bootstrapping typically involves creating new samples of the same size as the original by sampling with replacement. The authors' approach, generating one new sample (2.5k data points) much larger than the original (60 data points) by sampling from the estimated density function, is not appropriate. Additionally, instead of fitting a model to the data generated from a smoothened density function, fitting the mixture model directly to the original data should be considered.

*Response #2: We thank the reviewer for this great comment. Based on the reviewer’s suggestion here and the comment 3 below, the revised mixture model now has no bootstrapping or density smoothing. In the original manuscript, we tried to avoid the effects of multiple zeros on mixture model fitting by smoothing and sampling. These zero signals are from cell lines that have no ChIP-seq reads for a given CE. In the revised manuscript, we implemented an imputation strategy for zero values (detailed below at Comment 3) and fit the mixture model based on 60 data points. The model converges well with the imputation values for zeros. We observed highly reproducible results (over 98% of the 2,647,140 CE instances across 60 cell lines) for the predicted statuses of CEs compared to our original manuscript. We have revised all figures and text accordingly. Revised as recommended.*

3. The cause of zero values in the resulting normalized activity matrix for CEs requires clarification. Treating these zero values as missing data and applying appropriate imputation techniques may be a viable solution to this issue, obviating the need for incorrect bootstrapping strategies.

*Response #3: We implemented an imputation strategy for zero values in the revised model. As we fit mixture model “vertically” for each CE across 60 cell lines, the imputation strategy was implemented “horizontally” based on ChIP-seq signals across CEs for each given cell line. In detail, for a zero signal at a CE X in a cell line Y, we replaced it as the half of minimum signals for all non-zero CEs in cell line Y. We repeated this process for all zero signals at different CEs in different cell lines. We re-implemented the mixture model based on the imputation signals in the revised manuscript. As the reviewer expected, we now are able to remove the “bootstrapping” and smoothing processes with reproducible results observed. Revised as recommended.*

4. Figure 1A requires additional information. The methodology for conducting pairwise comparisons of SEs among the 60 cancer cell lines needs to be clarified. Details on the statistical tests employed, considering the limited replicates for each cell line, should be provided. Additionally, the number of identified CEs and the average number of CEs per SE may also be provided to improve clarity.

*Response #4: We have added more information both in the caption of Figure 1A and the Results section to clarify this, detailed below.*

*Pairwise comparisons of SE profiles were performed using software DASE (3), that was developed previously to capture different types of SE alterations including activity changes and constituent alterations. DASE statistically evaluated the CE patterns between cell lines by considering both the divergence between cell lines and the consistency of two replicates within each cell line. It reported differential SEs with statistical significance and differential types (e.g. activity or constituent change, etc.). Figure 1A shows the number of SEs that are altered (FDR <0.05 and at least one type of differential changes detected) and not altered between pairwise comparisons. Error bars are standard deviation across 1770 comparisons. The number of CEs in the pairwise analysis is 9530 per pair with an average of 7 per SE.*

*Revised as recommended.*

5. The section "Cancer/Cell-specific CE/SE identification" is challenging to comprehend. A plot illustrating this process could greatly enhance understanding. Additionally, the authors should provide further explanation and context for terms such as "prevalence threshold," "variation of information," and "inflection point."

*Response #5: We have added a chart to illustrate this process in Fig S3. We have added explanation in the main text for the three terms. Simple description of the three terms is also showed below.*

*Prevalence threshold was defined to select cell-specific CEs. In brief, a CE holding lower prevalence than the threshold will be determined as cell-specific. We selected the optimal prevalence threshold as 0.21. If a CE presents in less than 0.21*60 ~= 13 cell lines, we call it cell-specific. Variation of information is a metric to evaluate the similarity of two clustering results for the same groups of samples (7). The smaller the variation of information, the more similar the two clustering results are. We used this metric to evaluate the efficiency of cell line clustering based on cell-type-specific CEs (selected by the prevalence threshold) by comparing it with true cell cluster labels (28 cancer groups). Inflection point is where the slope of smoothed curve equal to -1 on the plot between variation of information and CE prevalence cutoff in Figure 4A.*

*Revised as recommended.*

6. Figure 2D demonstrates comparable outcomes between the mixture model-based approach and the traditional peak calling method. Given the statistical concerns with the mixture model approach and the similarity in results, it may be more beneficial to shift the focus towards the broader significance of investigating cancer-specific SEs at the constituent level. In my opinion, this constitutes a pivotal contribution, and the subsequent creation of the database (R package) presents a valuable resource for researchers in the field. Expanding on the functionality and features of the database would greatly enhance the impact of this study. This aspect merits further elaboration and emphasis in the manuscript.

*Response #6: We agree with the reviewer that the results of mixture model-based and peak-calling-based approaches are overall comparable (~ 88% agreement). We have revised our mixture model as described above based on the reviewer’s suggestions. With the revised model, we observed over 11% of CEs (90k CE instances) were estimated with differed statuses compared to peak-calling method. We believe it is worthy to present these refined statuses of CEs, especially when they are associated to differed regulatory activities to target genes (Figure 3). In the meantime, agreeing with the reviewer, we believe our R-package-based database will be most valuable to the research community of the field. In the revised Discussion, we added one example to interpret cancer-specific roles of CE signatures in gene regulation (Fig S12 and below). We expect more functional annotation will be added to our package as the next steps to understand SEs in cancers.*

*Revised as recommended.*

7. To enhance the reproducibility of the research, I recommend that the authors provide access to the code used for processing and normalizing ChIP-seq data, implementing the mixture model, identifying cancer/cell-specific SEs and CEs, and analyzing the data generated from independent sequencing assays. Sharing this code will not only allow for validation of the methodology but also empower other researchers to apply and build upon this valuable work. This practice aligns with best practices in computational biology and will significantly benefit the scientific community.

*Response #7: We have deposited all analysis code mentioned by the reviewer on GitHub:* [*https://github.com/tenglab/cSEAdb_plos_code*](https://github.com/tenglab/cSEAdb_plos_code) *. Revised as recommended.*

Minor:
1. Page 8: "shewed distribution" should be corrected to "skewed distribution."

*Corrected.*

2. Page 9: The last sentence on this page contains a grammatical error and requires revision.

*Corrected.*

3. Page 11: "POLR2A ChIA-PET sequencing ... thus allows us to ..." contains a grammatical mistake and should be revised.

*Corrected.*

4. Page 16: "is demonstrated with" should be changed to "demonstrates."

*Corrected.*

5. Data availability: The word "data" is plural; please ensure that the plural form of the verb is used.

*Corrected.*

6. Figure S1: "activate group" should be corrected to "active group."

*Corrected.*

**Reviewer #3:**

The authors summarize fingerprint SEs based on the cancer-specific statuses of their component active enhancers. Interestingly, they build an easy-to use R package to facilitate the query, exploration, and visualization of fingerprint SEs across cancers. The package will help us to get the specific epigenetic status of cancers and obtain more useful information about cancer biomarker.

I suggest the author list the code how to obtain the specific SE in pancancer. I have no questions about other issue.

*Response: We appreciate the overall positive comments and have deposited all analysis code on GitHub:* [*https://github.com/tenglab/cSEAdb_plos_code*](https://github.com/tenglab/cSEAdb_plos_code)

1.	Ghandi, M., Huang, F.W., Jane-Valbuena, J., Kryukov, G.V., Lo, C.C., McDonald, E.R., 3rd, Barretina, J., Gelfand, E.T., Bielski, C.M., Li, H. *et al.* (2019) Next-generation characterization of the Cancer Cell Line Encyclopedia. *Nature*, **569**, 503-508.

2.	Huang, J., Li, K., Cai, W., Liu, X., Zhang, Y., Orkin, S.H., Xu, J. and Yuan, G.C. (2018) Dissecting super-enhancer hierarchy based on chromatin interactions. *Nat Commun*, **9**, 943.

3.	Liu, X., Zhao, B., Shaw, T.I., Fridley, B.L., Duckett, D.R., Tan, A.C. and Teng, M. (2022) Summarizing internal dynamics boosts differential analysis and functional interpretation of super enhancers. *Nucleic Acids Res*, **50**, 3115-3127.

4.	Kai, Y., Li, B.E., Zhu, M., Li, G.Y., Chen, F., Han, Y., Cha, H.J., Orkin, S.H., Cai, W., Huang, J. *et al.* (2021) Mapping the evolving landscape of super-enhancers during cell differentiation. *Genome Biol*, **22**, 269.

5.	Sethi, A., Gu, M., Gumusgoz, E., Chan, L., Yan, K.K., Rozowsky, J., Barozzi, I., Afzal, V., Akiyama, J.A., Plajzer-Frick, I. *et al.* (2020) Supervised enhancer prediction with epigenetic pattern recognition and targeted validation. *Nat Methods*, **17**, 807-814.

6.	Mantsoki, A., Parussel, K. and Joshi, A. (2021) Identification and Characterisation of Putative Enhancer Elements in Mouse Embryonic Stem Cells. *Bioinform Biol Insights*, **15**, 1177932220974623.

7.	Meila, M. (2003) Comparing clusterings by the variation of information. *Lect Notes Artif Int*, **2777**, 173-187.
