# Supplementary material for: An Epigenomic fingerprint of human cancers by landscape interrogation of super enhancers at the constituent level
Source: PLoS Comput Biol. 2024 Feb 9;20(2):e1011873. doi: 10.1371/journal.pcbi.1011873 (PMC10883583; doi:10.1371/journal.pcbi.1011873)
Supplement: Attachment — Submitted filename: point-by-point response_final.docx [file pcbi.1011873.s017.docx]

*We thank the editor and reviewers for their further comments.*

Reviewer #1:

Minor Comments [1] :The authors have to describe in more detail the results of figure S7 and figure S8 in the corresponding legends.

*Response: We have added more detail to the legend of Figs S7 and S8. See below:*

*Figure S7.* ***Extra examples illustrating the improved sensitivity in detecting true active CEs by mixture models compared to peak calling****. (a). Red shaded square is an enhancer region under-estimated in A549 cell line by peak calling methods but identified as active by mixture model. This region shows strong enhancer activity in A549 compared to other cancer cell lines and presents regulatory interactions with target gene TRMT5 based on ChIA-PET data. (b). Similar to a) but for another enhancer region regulating SIX4 gene in A549 cell line. (c). Similar to a) but for another enhancer region regulating GNAI3 gene in HCT-116 cell line. (d). Similar to a) but for another enhancer region regulating HID1, MRPL58 and KCTD2 in HCT-116 cell line.*

*Figure S8.* ***Extra examples illustrating the improved specificity in detecting true inactive CEs by mixture models compared to peak calling****. Blue shaded square is an enhancer region over-estimated in A549 cell line by peak calling methods but identified as inactive by mixture model. This region shows weak enhancer activity in A549 compared to other cancer cell lines and presents no regulatory interactions with any genes based on ChIA-PET data. (b). Similar to a) but for another enhancer region in A549 cell line. (c). Similar to a) but for another enhancer region in HCT-116 cell line. (d). Similar to a) but for another enhancer region in MCF7 cell line.*

Figure Files: While revising your submission, please upload your figure files to the Preflight Analysis and Conversion Engine (PACE) digital diagnostic tool.

*Response: We have uploaded the figure files to PACE and converted the files to TIF format.*

Data Requirements: Please note that, as a condition of publication, PLOS' data policy requires that you make available all data used to draw the conclusions outlined in your manuscript. Data must be deposited in an appropriate repository, included within the body of the manuscript, or uploaded as supporting information. This includes all numerical values that were used to generate graphs, histograms etc..

*Response: All data to generate manuscript graphs and histograms are documented in Supplementary Data 1.*

Reproducibility: To enhance the reproducibility of your results, we recommend that you deposit your laboratory protocols in protocols.io, where a protocol can be assigned its own identifier (DOI) such that it can be cited independently in the future.

*Response: We have created a protocol documenting the computational framework of generating SE fingerprints for cancers using NCI-60 H3K27Ac ChIP-seq data. The protocol DOI is:* [*10.17504/protocols.io.kxygx38wzg8j/v1*](https://dx.doi.org/10.17504/protocols.io.kxygx38wzg8j/v1)*.*

References: Review your reference list to ensure that it is complete and correct.

*Response: There are no changes to the reference list. No retracted articles are cited. All references were reviewed. In the revised manuscript, we corrected typos for author names and page numbers for three references.*
